# Massive Cavitation by Pneumocystis jirovecii in an Immunocompromised Patient

**DOI:** 10.7759/cureus.25354

**Published:** 2022-05-26

**Authors:** Aldair Chaar-Hernandez, Jorge Montes, Maria C Rojas, Diego A Padilla-Mantilla, Abdelilah Lahmar, Juan F Toledo-Martinez, Francisco J Somoza-Cano

**Affiliations:** 1 Internal Medicine, Universidad de La Sabana, Bogota, COL; 2 Internal Medicine, Universidad de Los Andes, Bogota, COL; 3 Medicine, Faculty of Medicine and Pharmacy of Oujda, Oujda, MAR; 4 Internal Medicine, Cardio Center, San Pedro Sula, HND; 5 Internal Medicine, Universidad Católica de Honduras, San Pedro Sula, HND; 6 Internal Medicine, St. Vincent Charity Medical Center, Cleveland, USA; 7 Internal Medicine, Northeast Ohio Medical University, Cleveland, USA

**Keywords:** pneumocystis jirovecii pneumonia, cavitation, pulmonary cavitation, lung cavitation, hiv, aids, opportunist infections in hiv, medication noncompliance

## Abstract

Since the development of antiretroviral therapy (ART) and antibiotic prophylaxis, the incidence of opportunistic infections in human immunodeficiency virus-acquired immunodeficiency syndrome (HIV-AIDS) has been drastically reduced. However, third-world countries remain a fertile ground for medication nonadherence and inappropriate patient follow-up. Here, we present the case of a 42-year-old male with a history of HIV who presented with worsening shortness of breath and atypical chest pain. A chest X-ray and chest computed tomography scan revealed a left parahilar cavitation measuring 86 mm in diameter. A percutaneous lung biopsy revealed *Pneumocystis jirovecii*. Appropriate antibiotics were started, and the patient’s clinical status significantly improved. This case illustrates the devastating consequences of uncontrolled HIV-AIDS. ART and prophylactic antibiotics remain the cornerstone of treatment to ameliorate progressive lung damage in patients.

## Introduction

*Pneumocystis jirovecii* is an opportunistic microbe that is usually seen in patients with a compromised immune system [1]. It was previously classified as a parasite (protozoan), but in the 1980s, it was reclassified as an opportunistic yeast-like fungus [1-3]. To avoid confusion, *Pneumocystis jirovecii* pneumonia (PCP) is still abbreviated as PCP. Moreover, medical conditions that weaken the immune system, such as acquired immunodeficiency syndrome (AIDS), high-dose corticosteroids, immunosuppressants, or cancer, remain the main risk factors for PCP [3,4]. Patients with human immunodeficiency virus (HIV)-AIDS are less likely to get PCP in developed countries now that antiretroviral therapy (ART) is commonly available [1,4]. However, PCP remains a significant public health problem in developing countries [1,4,5]. Here, we present the case of a 42-year-old-male who was lost to follow-up after his HIV diagnosis and presented with massive lung cavitation secondary to *P. jirovecii*.

## Case presentation

**Figure 1:**
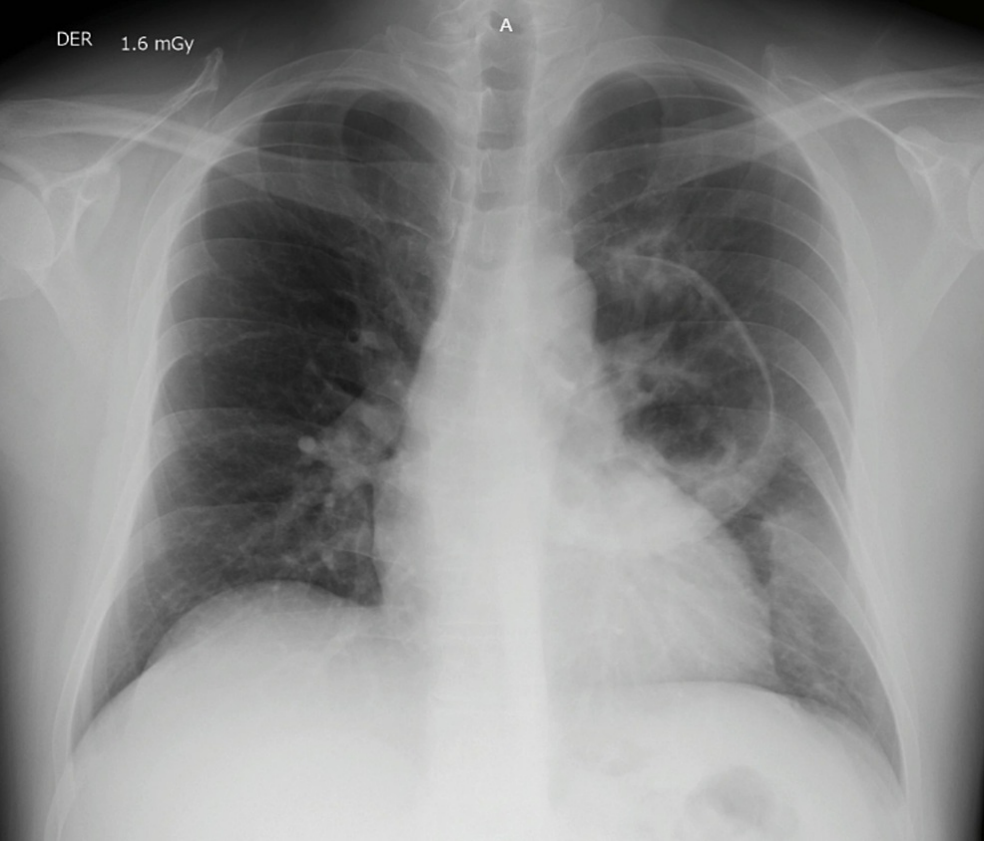
Anteroposterior chest X-ray. An anteroposterior chest X-ray was obtained in the emergency department upon initial presentation. A large left parahilar cavitation was observed.

**Figure 2:**
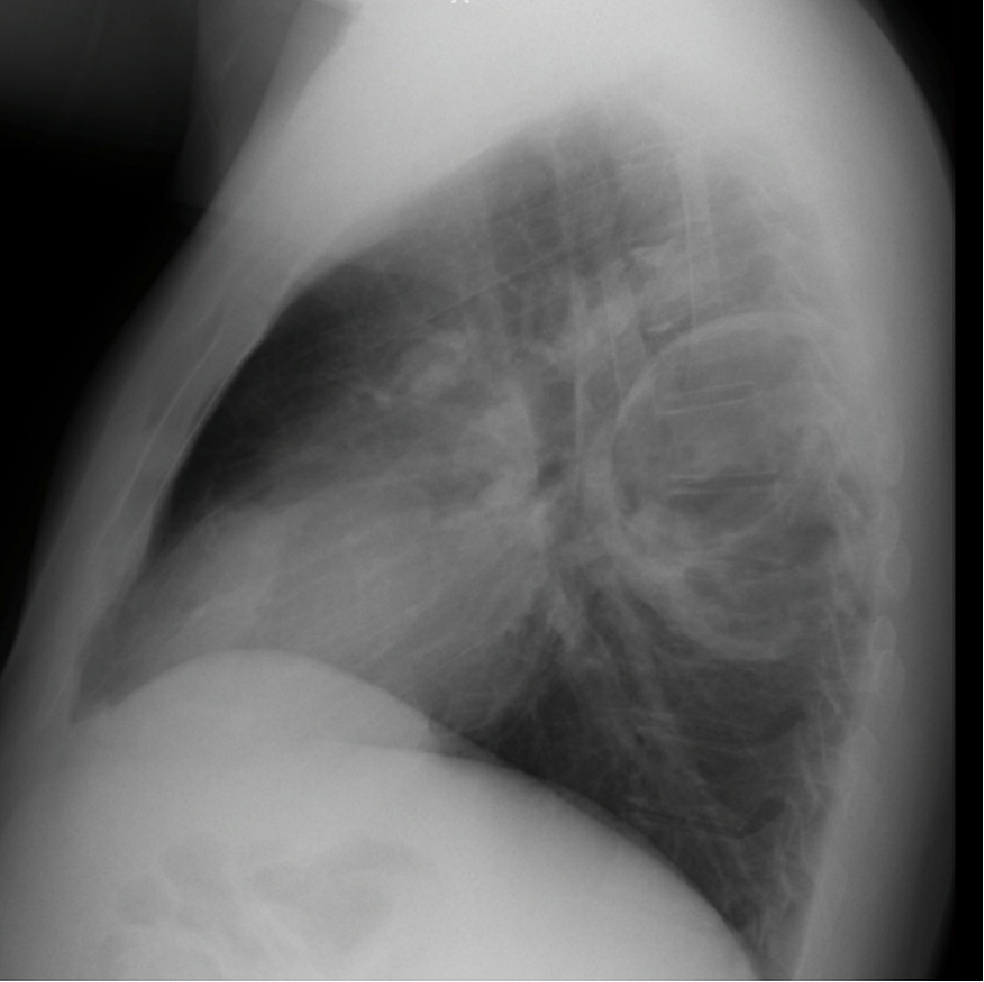
Lateral chest X-ray. A lateral chest X-ray confirmed a round lesion with defined borders on the left lower lung lobe.

A 42-year-old male with a medical history of HIV presented to the emergency department with a two-week history of dry cough, worsening shortness of breath, and noncardiac chest pain upon deep inspiration. His vital signs were stable except for a respiratory rate of 22 breaths/minute. Physical examination revealed diminished breath sounds in the upper left lung. The initial workup was grossly unremarkable except for a CD4 count of 150 cells/mm^3^. The β-d-glucan (BDG) test assay was negative. A chest X-ray showed a cavitary lesion with calcified walls in the left upper lobe (Figures 1, 2).

**Figure 3:**
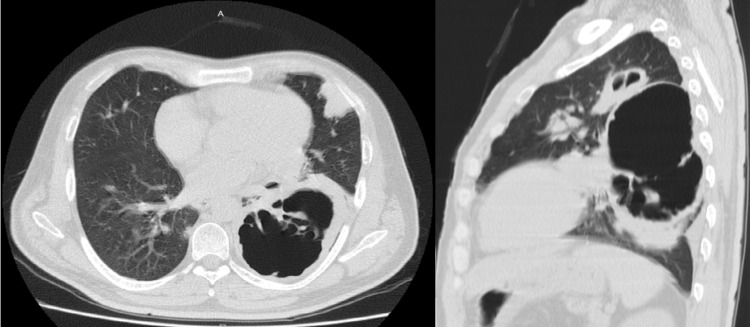
Chest computed tomography scan. A chest computed tomography scan in transverse (A) and sagittal views (B) showed left lower lung cavitation measuring 86 mm in diameter.

A chest computed tomography (CT) scan revealed a left parahilar, quistic lesion with septi, measuring around 86 mm in diameter (Figure 3).

A flexible fibrobronchoscopy with bronchoalveolar lavage was performed and gram-positive cocci and gram-negative bacilli were reported. A percutaneous lung biopsy of the lesion revealed *P. jirovecii* for which directed antibiotic therapy was started. Extensive workup for other opportunistic infections was negative. The patient was discharged on ART plus trimethoprim/sulfamethoxazole (TMP/SMX) with close monitoring for immune reconstitution inflammatory syndrome. His symptoms resolved thereafter.

## Discussion

*P. jirovecii* is an atypical fungus with a high prevalence of pneumonia in immunocompromised patients [1,4]. This fungus lives in the alveolar epithelium for which the alveoli’s macrophages are the first line of defense against this infection. However, with dysregulated cell immunodeficiency, this former parasite thrives and easily infects the lungs [1-4]. Once a cavitated lesion has been identified, it should prompt immediate clinical suspicion for this syndrome.

The annual incidence of PCP cases in Columbia is estimated at 1,525 (3.1 per 100,000 inhabitants) [5]. According to the study by Acevedo et al. conducted at Hospital La María de Medellin in Colombia, which enrolled 218 hospitalized patients, the incidence of* P. jirovecii* was 11.9%, and the prevalence of AIDS in this group of patients was 19.2% [6]. Due to the unrecognized HIV-positive status in many patients, *P. jirovecii* prophylaxis remains undertreated, which may further worsen the prognosis [7,8]. The incidence of PCP dates back to the 1980s when it was closely associated with the HIV epidemic in the United States and was considered as one of the major causes of death in the immunocompromised population. At its peak in 1990, it affected around 20,000 patients per year in the United States alone [9,10]. However, the number of cases of PCP decreased in HIV-positive patients after the ART and several public health measures were developed. This downtrend of the disease prevented many deaths as the mortality rate of patients admitted to the intensive care unit due to PCP surpasses 80% in certain patient cohorts [11,12].

*P. jirovecii* is mainly localized in the pulmonary alveoli of infected hosts. The trophic form attaches to type-1 alveolar epithelial cells through their filopodia, which promotes the proliferation of the microorganism and affects the growth of pulmonary epithelial cells. It is a simple apposition of cell surfaces without the fusion of membranes [12]. The adhesion of the fungus to the epithelial cells causes the recruitment of alveolar macrophages which are the main phagocytes involved in the elimination of *P. jirovecii* from the lungs as they express numerous receptors [12]. When alveolar macrophages are bound to molecules present on the surface of the pathogen, they cause the production of a large number of pro-inflammatory molecules. The mannose receptors of macrophages play a crucial role in the recognition of the major surface glycoprotein (MSG) and, thus, the uptake and elimination of the fungus [13]. It has been shown that an interaction between the dectin-1 receptor of the macrophage and the β-glucan of the wall of the microorganism, which plays a role in the generation of hydrogen peroxide, is involved in the elimination of *P. jirovecii* [12]. The inflammatory response is amplified by the production of pro-inflammatory chemokines and cytokines by alveolar macrophages and epithelial cells. Tumor necrosis factor-alpha (TNF-α) induces the production of interleukin-8 (IL-8) and interferon-gamma (IFN-γ). These mediators stimulate the recruitment and activation of lymphocytes, neutrophils, and monocytes [13,14]. T-CD4+ cells, via IFN-γ, coordinate the inflammatory response by recruiting and activating other immune effector cells, such as macrophages and monocytes, responsible for the elimination of *P. jirovecii *from the body. The role of CD8+ T lymphocytes is more controversial and they could have beneficial effects in situations of chronic depletion of T-CD4 cells [15]. Neutrophils, recruited by IL-8, are also involved in inflammation more than clearance. However, elevated neutrophils may be predictive of PCP-induced lung failure [12,15,16]. When CD4 counts are less than 200/mm^3^, patients infected with HIV are at high risk of developing pneumocystis [17,18].

PCP is characterized by a low-grade fever, nonproductive cough, and dyspnea. Less common symptoms include chest discomfort, weight loss, chills, and hemoptysis [17-19]. Asymptomatic cases are rare [17]. Although chest auscultation may be normal, one may detect diffuse discrete crackles; however, the clinical examination is usually nonspecific. The symptomatology, however, varies depending on the clinical context. In HIV-positive patients, the clinical picture is less acute and characterized by a triad of progressive onset of hyperthermia, nonproductive cough, and dyspnea of increasing intensity [17-19]. There may be pure febrile forms. As seen in this patient, PCP mainly occurs when the T-CD4 lymphocyte count is less than 200/mm^3^ [17-19]. In HIV-negative patients, the clinical picture is acute and severely complicated by acute respiratory failure. Furthermore, the duration of symptoms prior to diagnosis is shorter than that in HIV-infected patients, with a possible insidious and slow course, as described in HIV-positive cases [17]. Fortunately, the radiological presentation of PCP is the same regardless of the patient’s immunity status [20]. It is classically characterized by bilateral interstitial infiltrates, diffuse, fairly symmetrical, or granular (ground-glass) opacities, predominantly in the perihilar region or at the bases. The chest X-ray may appear normal, especially in the early stages. High-resolution CT scanning of the chest is more sensitive, allowing the diagnosis of PCP when the chest X-ray is negative [20].

Although the radiological aspects described above are typical of pulmonary infection with* P. jirovecii*, the existence of focal cystic lesions has been reported, with only a few studies showing cavitary lesions during PCP [21,22]. The definitive diagnosis of PCP is made by polymerase chain reaction (PCR) of respiratory specimens or fluorescein antibody staining [23]. Kaouech et al. performed a comparative study of PCR and staining techniques. They enrolled 54 immunocompromised patients with clinical symptoms of pulmonary infection and diagnosed PCP in 15 patients. Of the 15 patients, 14 were positive by PCR and only five were positive by techniques of staining, with a reported sensitivity and specificity of 93.3% and 87.1% for PCR and 33.3% and 100% for staining techniques, respectively. This explains the sensitivity of PCR and how it can be used to confirm a diagnosis, particularly in the case of superficial samples [23]. Furthermore, BDG has been studied and may be useful for diagnosing PCP in HIV patients with a sensitivity of 96% and a specificity of 86% [24].

The recommended treatment for PCP is the combination of TMP/SMX. This is effective for moderate-to-severe PCP, regardless of the HIV status due to its effectiveness over other regimens tested. It is generally recommended to be initiated in people infected with HIV when the CD4 cell count is below 200 cells/mm^3^ as a prophylactic measure. The main drawbacks of TMP/SMX are the side effects such as fever, aseptic meningitis, anaphylaxis, skin rashes, gastrointestinal complications, hepatotoxicity, pancreatitis, hyperkalemia, interstitial nephritis, renal insufficiency, marrow suppression, cytopenia, and the dreadful Steven-Johnson/toxic epidermal necrolysis syndrome [13,18,25].

In the case of an allergy to TMP-SMX, there are other medications that may be used. Clindamycin-primaquine is suggested for severely ill patients who are not eligible to take TMP-SMX. Unfortunately, several combinations have failed to demonstrate improvement. Trimethoprim-dapsone is not recommended for patients who have a serious reaction because it can be a fatal cause of the idiosyncratic dapsone-hypersensitivity syndrome. Moreover, due to the resistance mechanisms of* P. jirovecii*, there is evidence of prophylaxis failure with atovaquone, though it is better tolerated than TMP-SMX [18]. Furthermore, multiple studies have been done to specify the role of adjunctive corticosteroid treatment. HIV-negative PCP patients with hypoxemic respiratory failure improved clinically with better outcomes after steroids with a potential mortality benefit. However, the aforementioned therapy may be associated with an increase in mortality in HIV-negative PCP with non-hypoxemic respiratory failure [26,27].

PCP prevalence varies worldwide between 10% and 50% depending on the country. The death rate remains high in Africa and Latin America. Patients with a CD4 level lower than 200 cells/mm^3^ were more likely to present with the infection, up to 2.88, times compared with patients with CD4 counts above 200 cells/mm^3^. Additionally, the incidence of this infection in HIV-infected patients decreases with the use of ART and PCP prophylaxis [28]. Poor prognosis factors for PCP are partial oxygen pressure of less than 50 mmHg in room air, a major increase in serum lactate dehydrogenase, neutrophils greater than 10% in the BAL, and the presence of mutations in the *DHPS* gene. The prognosis also depends on age, adherence to therapy, delayed diagnosis, and co-infection by other pathogens [29].

## Conclusions

PCP must always be a top differential in immunocompromised patients, especially in those with a CD4 count of fewer than 200 cells/mm^3^ and cavitary lung lesions. Furthermore, ART and directed antibiotic therapy are essential to assure patient survival and reduce the morbimortality in this patient population.
